# New statistical potential for quality assessment of protein models and a survey of energy functions

**DOI:** 10.1186/1471-2105-11-128

**Published:** 2010-03-12

**Authors:** Dmitry Rykunov, Andras Fiser

**Affiliations:** 1 Department of Systems and Computational Biology, Albert Einstein College of Medicine, 1300 Morris Park Ave., Bronx, NY 10461, USA; 2 Department of Biochemistry, Albert Einstein College of Medicine, 1300 Morris Park Ave., Bronx, NY 10461, USA

## Abstract

**Background:**

Scoring functions, such as molecular mechanic forcefields and statistical potentials are fundamentally important tools in protein structure modeling and quality assessment.

**Results:**

The performances of a number of publicly available scoring functions are compared with a statistical rigor, with an emphasis on knowledge-based potentials. We explored the effect on accuracy of alternative choices for representing interaction center types and other features of scoring functions, such as using information on solvent accessibility, on torsion angles, accounting for secondary structure preferences and side chain orientation. Partially based on the observations made, we present a novel residue based statistical potential, which employs a shuffled reference state definition and takes into account the mutual orientation of residue side chains. Atom- and residue-level statistical potentials and Linux executables to calculate the energy of a given protein proposed in this work can be downloaded from http://www.fiserlab.org/potentials.

**Conclusions:**

Among the most influential terms we observed a critical role of a proper reference state definition and the benefits of including information about the microenvironment of interaction centers. Molecular mechanical potentials were also tested and found to be over-sensitive to small local imperfections in a structure, requiring unfeasible long energy relaxation before energy scores started to correlate with model quality.

## Background

Statistical potentials are widely used tools for protein structure analysis, modeling and quality assessment. Many different aspects and properties of these potentials have been explored during the last few decades including the different theoretical foundations to derive them, the representation of interaction centers and types of interactions, and the various models for defining the reference state. Combinations of various types and flavors of potentials are often used together in order to boost their performance. Initially, statistical potentials were based on statistical mechanics [1-3], however knowledge-based potentials now employ many other ideas, including the use of conditional probabilities to observe particular atom or residue distributions in specific conditions [4], linear programming techniques [5,6], linear and quadratic programming on various decoy sets [7], or information theory [8].

Despite the seemingly similar formalism to derive statistical potentials in general, the alternative definitions may result in very different performances. The majority of statistical potentials are pairwise potentials. In addition, single body potentials, like the ones accounting for solvent accessibility [9], were reported, as well as multibody potentials [10-12]. Although pairwise potentials are frequently used in combination with other types of potentials to improve their performance, multibody potentials are much less used, apparently due to the high computational cost to apply them. In the present work we focus on pairwise potentials.

The majority of statistical potentials employ the Boltzmann law to convert the observed frequencies of interactions into potentials. These potentials are obtained as the ratio of observed and expected frequencies, where the expected frequencies are derived from a hypothetical reference state when no interactions occur. While the observed interactions can be counted in experimentally solved structures, hypothetical protein models without interactions, which serve as reference states, are solely imaginary. Therefore depending on their actual design they are potential sources of great variability in the performance of statistical potentials.

Quasi-chemical approximation, a popular model for defining the reference state [2,13-16], uses molar fractions of the corresponding interacting centers to calculate the expected frequency of their pairs in the system without interactions and otherwise does not provide any other assumptions regarding their spatial distribution. This approach implies a homogeneous, infinite system, which of course is not true for proteins. An interesting attempt to account for the finite size of proteins was to substitute the corresponding dissipation of the atom density with a reduced effective dimensionality of the space [17,18]. "Isotropic" reference state, which is based on the occurrence of interacting pairs of any type at the given condition (distance, angle, etc.), appears natural and was also widely used [4,19,20]. However, it also approximates the system as infinite and homogeneous. A reference state that is free of these limitations was recently developed on a basis of shuffled systems [21] and a similar approach was suggested in the DOPE [22] potential. The reference state in the DOPE potential was defined as a homogeneous ensemble of non-interacting atoms in a sphere with the radius equal to the radius of gyration of a sample native structure, whereas our Shuffled Reference State model preserved spatial positions of the interacting centers in proteins, while their identities were shuffled. Further improvements to DOPE potential have been reported later [23]. Some other definitions of reference state, such as the use of decoys [5], were also suggested.

Different representations of interaction centers were explored in statistical potentials. Two major classes of explored representations are residue level or atomic. The residue level representations use C_α_, C_β _atoms or side chain centroids and are usually based on the 20 naturally occurring amino acids [2,15,24,25], although both reduced [7,26,27] and extended [28,29] amino acid alphabets were explored, where the extended alphabet further classifies each residue according to the possible secondary structure types. Another representation of interaction centers utilizes profile-based representation of amino acids residues [30]. For each protein a PSI-BLAST [31] generated alignment is used to create a position specific scoring matrix, which is converted into a set of evolutionary allowed amino acid residues for each position in the protein. Then these sets are used to derive potentials in a similar way to methods published by Melo [20] and Sippl [19]. Side-chain-to-backbone and side-chain to side-chain residue level potentials were also described [32]. All-heavy-atom representations based on reduced [20] and detailed all-atom protein representations were suggested [4]. More elaborate modifications of atomic alphabets consist of reduced set of atom types grouped by their chemical types and substitution states [33]. Micro-environments of atoms were distinguished by their chemical nature and by the counts of surrounding atoms. A potential function based on two interaction centers per residue [34] was also reported (all above examples employ one interaction center per residue). These two centers were C_α _atoms and the side chain center of masses (C_α _atom in the case of Glycine).

Various models of interactions were explored during the developments of statistical potentials. The most widely used ones are the distant-dependent potentials, which either treat all contacts uniformly within a cutoff distance [2,15,24,25], or account for their radial distribution [9,19-21,35-37]. Similar to the distance-dependent potentials are the contact area [38] and packing density potentials proposed by Li and Liang, (unpublished but available for download from http://gila.bioengr.uic.edu/resources/geometric.html). Another frequently used interaction model is based on angular dependence. Distributions of backbone ϕ, ψ torsion angles [3,32] as well as virtual κ, α angles [34,36] were explored. Promising combination of these degrees of freedom depends on both distance and orientation, which became more widely used recently [39-42].

Comparative analysis of contact potentials demonstrated that majority of them can be approximated by simple sum of amino acid hydrophobicities, while the rest depends on the hydrophobicities as well as on electrostatic properties [43].

In addition to the variety of ways to derive potentials, some additional techniques to improve their accuracy have been proposed. A trivial source of errors in statistical potentials is sparse statistics. Two major workarounds were developed: the use of pseudo-counts [4] and a weighting scheme suggested by Sippl [19]. Pseudo-counts simply add a unity to every count to avoid a division by zero when calculating fractions and do not try to normalize potential values in the case of empty counts, which could result in arbitrarily high positive values in certain cases. The weighting scheme assigns the average of all interaction types to the potential in the case of an empty count.

Composite potentials combine various terms, which may include solvation, residue-level pairwise, atomic level pairwise, hydrogen bonding, steric, torsion or secondary structure packing. One such example is the Rosetta scoring function [44,45]. Another, more recent example for a composite scoring function is QMEAN [28,46], which consists of six different terms: a torsion angle potential, secondary structure-specific, distance-dependent residue and all-atom pairwise potentials, a solvation potential as well as terms accounting for agreement of predicted and calculated secondary structure and solvent accessibility. A combination of mean force potentials, which account for distributions of pseudo-bonds, pseudo-angles, pseudo-dihedrals and distances between centers of interactions was studied [34]. Another composite potential, utilizing both residue-level (C_α_-based) [41] and its all-atom version [47] combines energy terms for distance-dependent pairwise interactions with orientation preference, hydrogen bonding, short-range interactions, packing, tri-peptide packing, three-body interactions, and solvation terms. Zhang and colleagues proposed a composite residue-level potential that consists of contact and local energy terms and employs a reduced alphabet of amino acids and a mapping of protein structures into a discrete state model [48]. The potential was generated by optimizing its components in order to guarantee a minimum energy gap between the native and decoy structures in a training set.

In the present work we perform a systematic comparison of many of the above listed scoring functions using a large and diverse decoy set that is based on models collected during various CASP experiments [49]. We analyze the differences in their performances of ranking protein models as a function of various flavors of scoring functions. Partially based on these results, we developed a novel residue level statistical potential that takes advantage of our earlier developed shuffled reference state definition [21] but utilizes orientation-dependent accounting for residue interactions. We demonstrate that this novel potential is highly competitive with other scoring functions.

## Results and Discussion

### Benchmarking potential functions

Evaluating the performance of various statistical potentials using protein-like decoys is not a trivial problem. Decoys must present a balanced range of difficulty or be specific for a particular task or property [50]. Some scoring functions identify the native structure easily among a set of decoys but perform very poorly when it comes to identifying the most accurate model from the rest of the decoys in the absence of the native structure. This can happen because of overtraining on native structures or because of significant structural differences between the decoys and the native structure. As a consequence, benchmarks that include the native structure in the decoy set may not be informative or challenging enough for most scoring functions. On the other hand, a decoy set without a native structure has its own limitations because it is not guaranteed that a decoy with the highest geometrical similarity to the native structure (e.g. lowest root mean square deviation) is also the one with the lowest energy. The model that is most similar to the native structure might have a higher energy due to some locally unfavorable features. Nevertheless, this approach seems more practical because scoring functions are typically used in scenarios when the native structure is not known and only a variety of possible alternative models are available.

Another problem arises when only one method or a limited number of methods is used to generate decoys, which is often the case for other available decoy sets [51-53]. In these cases a scoring function might be specific to implicit features of the decoy generation procedure but perform significantly worse if used to score decoys of different origin. These potential problems can be avoided with the use of a large number of targets in a decoy set and by a careful selection of decoy properties, such as using standardized similarity to the native structure and using a diversity of methods to generate decoy models.

In the present work, we tested scoring functions on decoys with and without the native structure, emphasizing on the latter set. "Global distance test - total score" (GDT_TS, which is (GDT_P1 + GDT_P2 + GDT_P4 + GDT_P8)/4, where GDT_Pn denotes percent of residues under distance cutoff ≤ nÅ) values [54,55] were used to assess structural similarity of decoy models to the corresponding experimental solution structure of the target. Scores were binned in 2.5 GDT_TS units (i.e. models that are less than 2.5 GDT_TS units different from each other were considered indistinguishable), and bin numbers were used as rank values starting from the highest GDT_TS value. This scheme makes sets of decoys of different quality comparable to one another. Although the choice of 2.5 GDT_TS units for binning is subjective, any other value would be subjective to the same extent. Meanwhile this value provides enough granularity for a statistical survey, while groups together essentially indistinguishable models. However, when native structure is included in a decoy set, this approach may over-penalize mispredictions. The GDT_TS score of a native structure is 100 by definition and, according to the selection process, the closest model can be as low as 65 (see Methods for details). Therefore, if the native structure is included in the test set, it may be separated from the most accurate decoy model by a significant accuracy gap, up to 14 bins. Consequently, misrecognition of the native model, when it is included in the set, is heavily penalized. To overcome this effect, we always assign the rank of 2 to the first non-native structure, if the native one is present, regardless of the number of empty bins separating them.

### Impact of different protein representations on performance

According the representation of interaction centers used scoring functions evaluated in this study can be classified in three major groups: (i) atom-based, i.e. all-heavy-atom or reduced set of atom types, namely "QMEAN-all_atom" [46], "OPUS_PSP" [47], "DOPE" [22], "dFIRE" [17], "Shortle2006" [35], VSCORE-pair [38], ANOLEA-like ("Melo-ANOLEA", "Melo-NL") [56], our "RF_HA", "RF_HA_SRS" [21], and "Liang-geometric" potentials, (ii) residue-based: "QMEAN-pairwise", "QMEAN-SSE_agree", "QMEAN-ACC_agree", "QMEAN-torsion", "QMEAN-solvation" [28], "Floudas-Ca" [5], "Floudas-CM" [6], "Dong-pair" [30] potentials, as well as potentials proposed in this work, "RF_CB_SRS_OD", "RF_CB_OD", "RF_CB_SRS", "RF_CB", and (iii) composite potentials: "PROSA-pair", "PROSA-combined" [57], "Rosetta" [44], "Shortle2005" [32], "QMEAN6" [28,46], "OPUS_CA" [41], "VSCORE-combined" [38], and "PC2CA" [34]. Composite potential functions most often are defined as a linear combination of residue-based long-range potentials with different kinds of local potentials, which are in most cases residue-based as well. In addition to the knowledge based scoring functions, a molecular mechanics potential, CHARMM [58], as implemented in the NAMD [59] package, was also evaluated. In terms of protein representation CHARMM can be categorized as a composite all-atom potential. Models were evaluated after subjecting them to one or 1000 relaxation steps, indicated as "NAMD 1" and "NAMD 1000", respectively.

The results of the benchmarking survey of the scoring functions are shown in Table 1. Data are sorted by the average rank of the lowest energy decoy structure in the absence of the native structure. It is noticeable that the performance of different potentials varies significantly depending on the presence or absence of the native structure. In the presence of a native structure all-atom potentials are usually more sensitive (i.e., the RF_HA_SRS and the Shortle2006 potentials are the top two). Meanwhile, no interaction type preference is observed if the native structure is absent from the test set: residue or atom based or composite potentials all perform competitively. In addition, potentials with good performances in the presence of the native structure often exhibit rather mediocre performance if the native structure is removed from the decoy set. For instance RF_HA_SRS, our all-atom potential with shuffled reference state definition [21], is the best performing potential recognizing the native structure correctly in 137 out of 143 decoy sets but ranks only as the 6^th ^best when tested on a set without the native structure (Table 1). Similarly the Shortle2006 potential, which is the second best recognizing the native structure among decoys ranks only 23^rd ^among potentials when the native structure is removed. This may indicate that atomistic potentials are often over-trained to recognize native structures or, alternatively, it may indicate that side-chain placement by current modeling methods is not accurate enough. Indirect support for the former hypothesis is the observation that reduction in number of atom types by joining chemically equivalent but distinct by PDB nomenclature types like Phe-CD1 and Phe-CD2 atoms into one Phe-CD type results in the loss of potential performance (data not shown).

**Table 1:** Performance of various statistical potentials on models of CASP5-8 experiments.

Scoring function	models only		native included		
	Average^a^	Ranked 1^b^	Average^c^	Raw average^d^	Ranked 1^e^
QMEAN6	2.87	85	1.71	3.26	113
QMEAN-all_atom	3.59	74	1.71	2.9	119
QMEAN-SSE_agree	3.74	62	3.72	9.62	39
QMEAN-ACC_agree	4.04	40	3.78	8.83	48
RF_CB_SRS_OD	4.16	61	2.08	3.6	110
RF_CB_OD	4.62	62	2.00	3.65	111
RF_HA_SRS	4.65	49	1.38	1.66	137
RF_CB_SRS	4.72	56	2.18	3.46	114
OPUS_CA	4.72	79	5.13	9.93	55
VSCORE-combined	4.79	53	2.20	3.79	117
QMEAN-pairwise	4.80	54	3.15	5.86	85
Rosetta	5.01	57	4.09	8.03	68
Dong-pair	5.01	58	6.32	14.41	4
RF_CB	5.06	52	2.46	4.31	106
VSCORE-pair	5.08	54	1.85	2.81	128
PROSA-combined	5.11	57	3.38	6.27	87
OPUS_PSP	5.39	54	2.99	4.11	118
RF_HA	5.44	62	2.78	4.37	112
DOPE	5.77	54	3.27	5.97	95
dFIRE	6.03	50	5.69	11.8	33
PROSA-pair	6.03	56	3.54	6.02	95
QMEAN-torsion	6.71	45	3.24	4.66	114
Shortle2006	6.85	35	1.79	2.54	129
Liang_geometric	6.88	44	2.48	3.94	114
QMEAN-solvation	7.32	33	6.27	10.87	54
Shortle2005	7.73	42	3.39	5.19	109
Floudas-CM	7.75	38	7.05	12.77	42
Floudas-Ca	7.79	33	8.36	16.01	10
NAMD 1000	8.06	24	4.96	8.56	78
Melo-ANOLEA	9.62	19	5.19	8.37	86
PC2CA	9.75	19	5.06	8.35	85
Melo-NL	9.99	14	5.85	9.45	80
NAMD 1	11.91	5	10.98	18.04	24

Random^¶^	9.72	13.9	10.1	10.1	8.3

^a ^The average rank (over 143 decoy sets) in the absence of native structures.

^b ^The number of sets when the best model was ranked as first, in the absence of native structures.

^c ^The average rank when native structures are present.

^d ^The average rank when native structures are present, calculated without compensation for the gap in ranking between experimental structure and first model (see text).

^e ^The number of sets when the best model was ranked as first when native structures are present.

^¶^Expected random values were generated by picking a wining model from the decoy sets randomly. Average values over 1000 random trials are shown.

Influence of different properties of scoring functions in test cases where the native structures are absent from the set of decoys is not as straightforward as it is in the case when the native structures are present. There is not a specific group of potentials that outperform others. The composite potential QMEAN6, with its individually evaluated all-atom term and components accounting for secondary structure and solvent accessibility agreement, is among the best performing potentials. The residue level RF_CB_SRS_OD potential proposed in the present work compares competitively in this test. However, QMEAN "agreement-based" terms perform rather modestly in the presence of the native structure, and all other functions discussed here (QMEAN6, QMEAN-all_atom and RF_CB_SRS_OD) underperform some other all-atom potentials (RF_HA_SRS and Shortle2006), as mentioned above.

### Assessing statistical significance of performance differences

**Figure 1:**
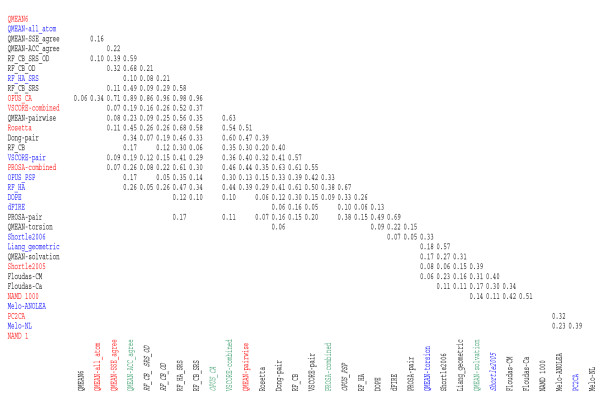
**Statistical significance of rank distributions obtained with different methods**. Only p-values obtained with one-tailed Wilcoxon test above 0.05 are shown. Alternative hypothesis in the test was that location of the distribution obtained with scoring function in a row is lower than the one in the column. Interaction centers are color coded in row titles as follows: black is used for residue-based potentials, blue is used for atom-based potentials, and red designates composite scoring functions. Certain interaction types are color coded in column titles, specifically, scoring functions making use of solvent accessibility (green), torsion angle dependence (blue), secondary structure dependence (red), and orientation-dependence (in italic).

An important question in benchmarking various potentials is the assessment of the statistical significance of differences of their performances. We performed pairwise one-tailed Wilcoxon tests on results obtained in the absence of the native structure (Fig. 1). Potentials are sorted in the same order as in Table 1. Only p-values higher than 0.05 are shown, pointing out pairs of scoring functions that are not significantly different from one another. We employed the Wilcoxon test because the distributions of the calculated ranks of decoys that scored as best are highly different from normal. In this test the null hypothesis is that the ranks calculated by two methods under comparison share the same distribution and the one-sided alternative is that the ranks obtained with the method listed in the row of the Fig. 1 are lower than ones obtained with the method listed in the column.

### The importance of reference state definition

A large group of various potentials, specifically QMEAN residue-based pairwise and accessibility agreement terms (QMEAN-SSE_agree, QMEAN-ACC_agree, QMEAN-pairwise), our atomic level potentials with shuffled (RF_HA_SRS) and classic (RF_HA) reference states, our residue-level potentials with and without orientation dependence and shuffled reference state (RF_SRS_CB_OD, RF_CB_SRS, RF_CB_OD, RF_CB), Rosetta++ scoring function (Rosetta), both pairwise (VSCORE-pair) and composite (VSCORE-combined) versions of VSCORE potential, PROSA2003 composite scoring function (PROSA-combined), as well as profile based distance-dependent potential from Dong group (Dong-pair) and OPUS_PSP potentials do not demonstrate statistically significant difference to one another (Fig. 1). However, one can speculate that p-values obtained for the residue-level regular (RF_CB_SRS) and orientation dependent (RF_CB_SRS_OD) potentials, both of which utilize a recently introduced shuffled reference state definition [21], are superior to (RF_CB), which is a potential based on a classic reference state definition (p-values of differences are 0.06 and 0.007, respectively). Orientation dependence is another important factor, which contributes significantly to the potential performance, resulting in statistically significant superiority the (RF_CB_SRS_OD) potential over classic reference state potential (RF_CB). It is also interesting to mention that the distribution of ranks obtained with OPUS_CA scoring function is located significantly lower on the rank scale than most other potentials in this group, whereas the average rank value calculated with this potential is in the middle of this group. This fact can be explained by the observation that OPUS_CA is able to score decoys with the highest GDT_TS values as the best ones in many more cases than other potentials in this group (Table 1). However, the relatively low average rank for this potential is because it exhibits a drastically high error in cases in which it fails to find the best structure.

The performance of the molecular mechanics based CHARMM potential depends on the number of steps of structure relaxation. The performance of the CHARMM is close to random after one and even after 1000 steps of relaxation. A further 10-fold increase in the energy minimization steps brings CHARMM performance to the middle of the group of similarly performing potentials, discussed above (average rank in the absence of native structure is 5.27, data not shown in the Table 1 and Fig. 1). However, the exceedingly high computational cost makes the use of such long minimizations impractical.

### Effect of accounting for microenvironments on the performance

It is interesting to survey the common features among the best performing potentials. As we noted above, the choice of the type of interaction center (either atomistic or residue level potential or a composite scoring function) does not correlate with the performance. Indeed, one can see, from the color coding of interaction center types in raw titles (Fig. 1), that potentials of every kind can be found over the entire range of performances. The very small number of residue level potentials that are based on interaction centers other than C_β _atoms (Floudas-Ca, Floudas-CM, PC2CA) does not allow us to draw a conclusion about their performance. Meanwhile, some conclusions can be drawn from the effect of certain other features of scoring functions, such as the use of solvent accessibility, torsion angle, accounting for secondary structure and consideration of orientation dependence. The aforementioned features are color-coded in the column titles of Fig. 1. The secondary structure dependent functions (red) perform better than average, whereas torsion angle dependent functions (blue) perform worse than average. Potentials using information on solvent accessibility (green) and orientation dependence (shown in italic) do not show a clear advantage.

**Figure 2:**
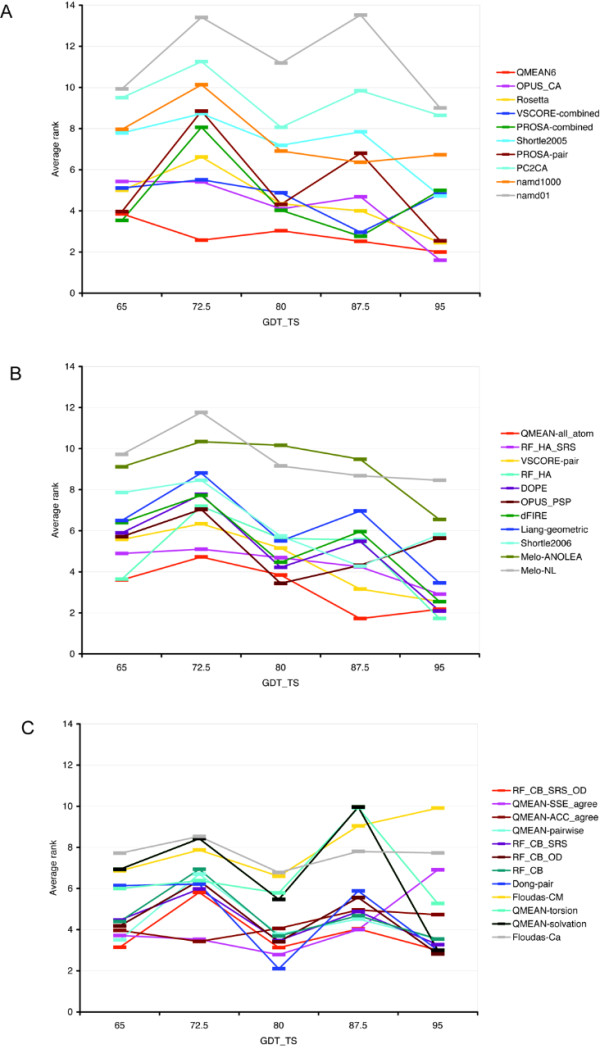
**Performance of different potentials as a function of the quality of the best available model**. Average rank calculated by (A) composite functions, (B) heavy-atom based functions, and (C) residue-based potential functions for targets having best model with GDT_TS better than 95.0 (11 targets), between 87.5 and 95.0 (25 targets), between 80.0 and 87.5 (32 targets), between 72.5 and 80.0 (47 targets) and between 65.0 and 72.5 (28 targets).

It is interesting to see if the performance of various scoring functions varies with the quality of the best available model for a given target. This dependence is plotted in Fig. 2, panels (A) (B) and (C) display the accuracy dependence of composite, all-atom and residue-based potentials, respectively. One can observe a general trend in the case of composite (Fig. 2A) and especially of all-atom potentials (Fig. 2B), according to which the performance improves with the improvement of the quality of the best available model. Noticeable exceptions are composite VSCORE and PROSA potentials, which perform visibly worse for the highest accuracy groups, when the best model has GDT_TS 95.0 or higher. These two potentials include solvent accessibility term in addition to their distance dependent terms. Solvent accessibility term may have limited benefit at this high accuracy level, when solvent accessibility of alternative models is essentially identical. Another example of such "reversed" dependence is OPUS_PSP, which is the best in the group of targets in the bin of 80.0, but its performance decreases as higher quality models become available. The group of residue-based potentials (Fig. 2C) does not show the above trends. Instead, this group collectively shows inferior performance for targets in 72.5 bin as compared to the 65.0 bin, as well as for targets in 87.5 bin as compared to 80.0 bin. An interesting exception is the performance of QMEAN-SSE_agree (using secondary structure dependent term) and QMEAN-ACC_agree (using solvent accessibility dependent term) potentials. Both are among the best ones for sets of targets with lower quality (65 and especially 72.5 bins), QMEAN-SSE_agree keeps its leading position up to 87.5 GDT_TS target group but looses its sensitivity as nearly perfect models of GDT_TS 95.0 or higher become available. This observation together with the outstanding performance of the QMEAN-all_atom potential, which is also a secondary structure dependent one, confirms the previous observations about the general benefit of incorporating secondary structure information in the potential function. However, the QMEAN-ACC_agree potential with solvent accessibility term loses its sensitivity much earlier. This behavior of the QMEAN-ACC_agree potential is in agreement with earlier discussed behavior of composite VSCORE and PROSA potentials, which also dependent on solvent accessibility.

We also reviewed the performance of the scoring functions as a function of various structural classes. Because only 56 out of 143 targets are currently classified in the SCOP database [60,61], the significance of such analysis is limited. We could not find a significant correlation between particular scoring function features and the fold classes (Additional file 1, Fig. A1). In general, all scoring functions show a better performance in case of α/β proteins, an average performance can be observed for all-α proteins and α+β proteins, while the worst performance is detected for all-β proteins.

## Conclusions

In summary, the correct definition of the reference state used in statistical potentials is critical. In addition, there seems to be a benefit of including information on various protein microenvironments. An effective reference state definition should be free of systematic errors, as it is in our SRS model, and actual interactions should be a function of amino acid frequency variations caused by local microenvironments such as different secondary structure preferences, and other deviations of local characteristics from the average.

## Methods

### Set of proteins and parameters used to derive residue-level potentials

A novel distance dependent residue-level potential (RF_CB_SRS_OD), utilizing shuffled reference state [21] and featuring orientation dependence, was derived from a representative set of 375 globular proteins selected from the Protein Data Bank[62]. General procedure and details for the protein set selection are described previously for all-heavy-atom potential [21]. Briefly, the set was composed of X-ray solved structures of proteins of at least 50 residues long, which crystallographic resolution and R-value were better than 2.1 Å and 0.2, respectively; all PDB structures with incomplete, missing, modified, or nonstandard residues were excluded except structures that had missing residues in the terminal positions only; structures co-crystallized with ions were also discarded; additionally, the pairwise sequence identity between any two proteins in the set was required to be less than 40%.

Three additional potentials were generated in order to evaluate its improvement over the "isotropic" reference state and over unidirectional accounting for interacting pairs. Two of these additional potentials employed averaging over all residue types [19], where one was built as orientation-dependent (RF_CB and RF_CB_OD, respectively). A third potential (RF_SRS) was based on shuffled reference state, but lacked the orientation dependence. For all of the potentials the first bin for spatial separation spanned the distance between 0-4 Å and every next bin spanned a 1 Å increment thereafter. C_β _atoms were used for system representation. A virtual C_β _atom was built for Glycine residue. No minimal sequence separation between interacting residues was required. For sparse data treatment the scheme introduced by Sippl [19] was used.

In order to generate a shuffled reference state, randomized model sets were obtained by shuffling residue identities within each protein. Shuffling procedure was repeated 1000 times using different seed values for the random number generator. Potentials were derived as described previously [21]

**Figure 3:**
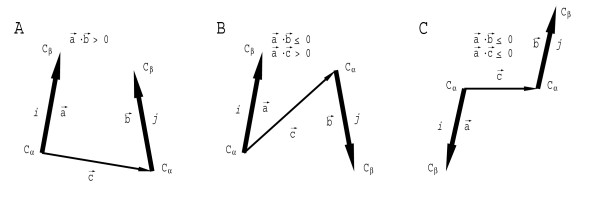
**Definition of residue orientation used to derive potentials**. (**A**) interaction *i *→ *j *is considered "parallel" if the scalar product of C_α_-C_β _vectors for residues *i *(vector **a**) and *j *(vector **b**) is positive; (**B**) interaction *i *→ *j *is considered "antiparallel *i *facing *j*" if scalar product of **a **and **b **is non-positive and scalar product of vector **a **and vector from C_α _atom of residue *i *to C_α _atom of residue *j *(vector **c**) is positive; (**C**) interaction *i *→ *j *is considered as "antiparallel *i *pointing away from *j*" if both **a**·**b **and **a**·**c **scalar products are non-positive.

For orientation-dependent potentials residue pairs were classified into three groups (Fig. 3): pairs with "parallel" C_α_-C_β _vectors, pairs with "antiparallel" C_α_-C_β _vectors facing each other, and ones with "antiparallel" C_α_-C_β _vectors pointing away from each other. This definition of the orientation dependence is substantially simplified in comparison to ones suggested earlier [39,42]. However, this simplification results in more representative statistics of contacts.

Potentials developed in the present study are labeled as "RF_CB", with "_SRS" suffix for shuffled reference state and "_OD" suffix for the orientation dependence.

### Set of decoys

**Figure 4:**
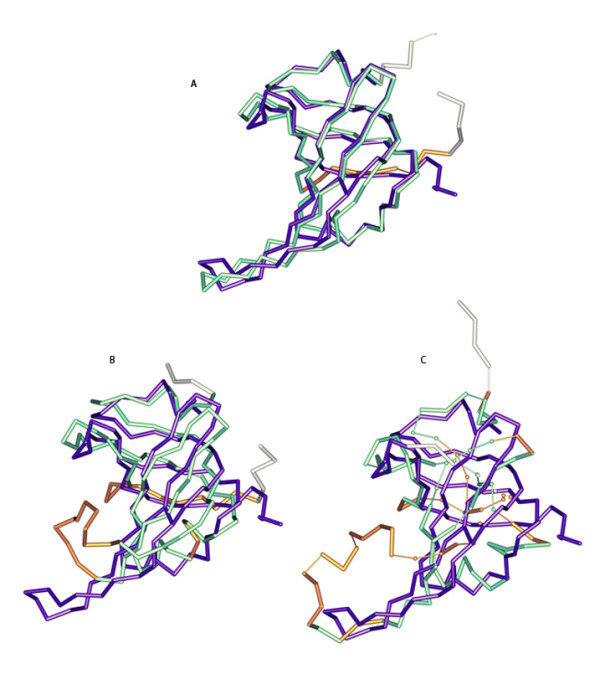
**Superimposition of models of different quality with the experimental solution structure**. Experimental structure of the CASP8 target T0502 (violet) and its models, (A) METATASSER_TS5, GDT_TS = 80.357, (B) 3Dpro_TS4, GDT_TS = 60.204, and (C) panther_server_TS2, GDT_TS = 44.643 are shown as C_α _traces. Those parts of the models, where the experimental positions are not known are colored white. Parts of models deviating from experimentally determined positions less than 4 Å are colored green, and the rest is colored bronze. C_α_-C_α _pseudobonds longer than 3.9 Å are shown thin. This plot has been generated using MOLSCRIPT software [63].

Predicted models for 143 targets collected from the CASP5-CASP8 experiments [49] were used as decoys, including a total of 2628 models produced by a large variety of groups and methods. These models were selected using the following procedure: (i) only all-atom models were used; (ii) the set for a given target was required to include at least one model with GDT_TS score upon superposition to the experimental solution structure 65.0 or better; (iii) all models for each target were clustered by their lengths, and models from the most populated cluster were used; (iv) models were binned by their GDT_TS scores with increments of 2.5 and one random representative was kept from each bin. Only targets for which the experimental solution is publicly available were kept. As a result, a ranked list of representative models was selected for each of the 143 targets. Fig. 4 gives an example of superimposition of models of different quality to the experimental structure. Lists of selected targets and their models along with corresponding GDT_TS values can be downloaded from our website http://www.fiserlab.org/potentials/casp_decoys

## Authors' contributions

DR and AF conceived and designed the study and wrote the manuscript. DR carried out the calculations. All authors read and approved the final version.

## Supplementary Material

Additional file 1 **Figure A1.** Performance of different potentials as a function of SCOP class definitions. Average ranks were obtained for target structures of specific SCOP classes using various scoring functions. Connecting lines facilitate visual tracking of results for a given scoring function. Click here for file
