# Supplementary figures and images for: New statistical potential for quality assessment of protein models and a survey of energy functions
Source: BMC Bioinformatics. 2010 Mar 12;11:128. doi: 10.1186/1471-2105-11-128 (PMC2853469; doi:10.1186/1471-2105-11-128)

Figure A1. Performance of different potentials as a function of the SCOP class.

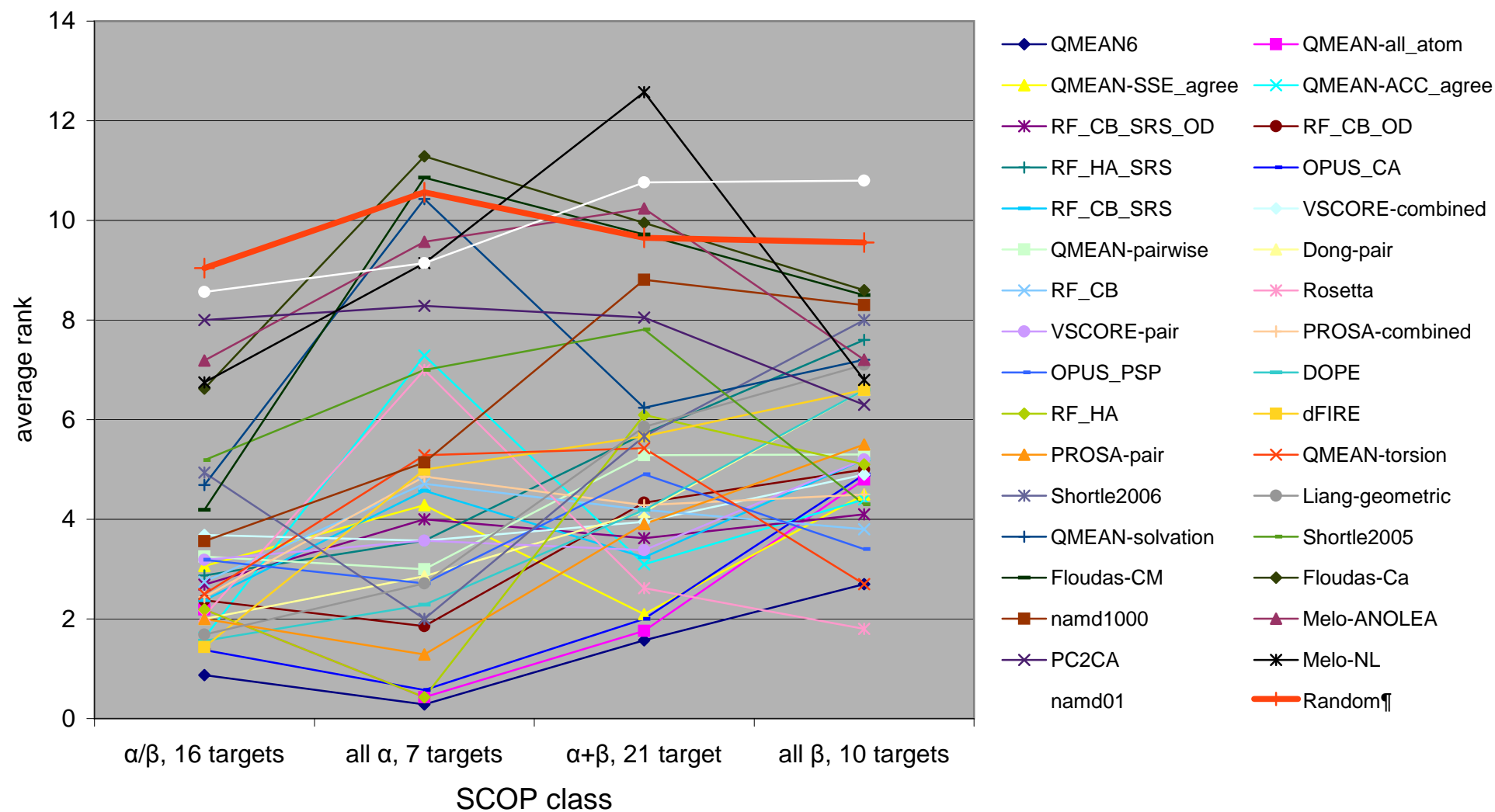

Supplement: Additional file 1 — Figure A1. Performance of different potentials as a function of SCOP class definitions. Average ranks were obtained for target structures of specific SCOP classes using various scoring functions. Connecting lines facilitate visual tracking of results for a given scoring function. [file 1471-2105-11-128-S1.PDF]
